# The accuracy of a designed software for automated localization of craniofacial landmarks on CBCT images

**DOI:** 10.1186/1471-2342-14-32

**Published:** 2014-09-16

**Authors:** Shoaleh Shahidi, Ehsan Bahrampour, Elham Soltanimehr, Ali Zamani, Morteza Oshagh, Marzieh Moattari, Alireza Mehdizadeh

**Affiliations:** 1 Biomaterial Research Center, Department of Oral and Maxillofacial Radiology, School of Dentistry, Shiraz University of Medical Sciences, Shiraz, Iran; 2 Department of Oral and Maxillofacial Radiology, School of Dentistry, Shiraz University of Medical Sciences, Shiraz, Iran; 3 Department of Pediatric Dentistry, School of Dentistry, Shiraz University of Medical Sciences, Shiraz, Iran; 4 Medical Physics and Medical Engineering Department, School of Medicine, Shiraz University of Medical Sciences, Shiraz, Iran; 5 Private Practice, Tehran, Iran; 6 Faculty of Nursing and Midwifery, Shiraz University of Medical Sciences, Shiraz, Iran

## Abstract

**Background:**

Two-dimensional projection radiographs have been traditionally considered the modality of choice for cephalometric analysis. To overcome the shortcomings of two-dimensional images, three-dimensional computed tomography (CT) has been used to evaluate craniofacial structures. However, manual landmark detection depends on medical expertise, and the process is time-consuming. The present study was designed to produce software capable of automated localization of craniofacial landmarks on cone beam (CB) CT images based on image registration and to evaluate its accuracy.

**Methods:**

The software was designed using MATLAB programming language. The technique was a combination of feature-based (principal axes registration) and voxel similarity-based methods for image registration. A total of 8 CBCT images were selected as our reference images for creating a head atlas. Then, 20 CBCT images were randomly selected as the test images for evaluating the method. Three experts twice located 14 landmarks in all 28 CBCT images during two examinations set 6 weeks apart. The differences in the distances of coordinates of each landmark on each image between manual and automated detection methods were calculated and reported as mean errors.

**Results:**

The combined intraclass correlation coefficient for intraobserver reliability was 0.89 and for interobserver reliability 0.87 (95% confidence interval, 0.82 to 0.93). The mean errors of all 14 landmarks were <4 mm. Additionally, 63.57% of landmarks had a mean error of <3 mm compared with manual detection (gold standard method).

**Conclusion:**

The accuracy of our approach for automated localization of craniofacial landmarks, which was based on combining feature-based and voxel similarity-based methods for image registration, was acceptable. Nevertheless we recommend repetition of this study using other techniques, such as intensity-based methods.

## Background

Cephalometric analysis is one of the key tools for arriving at an accurate diagnosis, planning treatment, evaluating growth, and research [1-3]. Landmark-based analysis is the most common method for cephalometric analysis [4]. Detection of landmarks plays an essential role in medical diagnosis and treatment planning [5].

Two-dimensional (2D) projection radiographs have been traditionally considered the modality of choice for orthodontic cephalometric analysis [6]. However, plain radiography has many shortcomings, such as superimposition of structures of the left and right sides of the skull, unequal magnification of bilateral structures [4], the possibility of distortion of mid-facial structures [7], and random errors that arise as a result of variations in positioning the patient in the cephalostat [8].

To overcome these shortcomings, three-dimensional (3D) computed tomography (CT) has been used to evaluate craniofacial structures with less distortion than plain-film views [4]. The introduction of cone beam computed tomography (CBCT) during the past decade offers advantages over plain CT, such as smaller machines, reduced costs, and increased accessibility [9]. With the development of CBCT, 3D assessment of the craniofacial region has become an alternative for patient imaging [10].

The accuracy and reliability of landmark detection and cephalometric measurements using 3D data gathered using the CBCT technique have been confirmed [1,4,9,11,12]. For example, it has been shown that 3D images are more accurate and reliable than traditional cephalographic projections for both landmark detection [4] and measurements [11]. However, using 3D landmark identification is more time-consuming than using conventional 2D cephalographic tracings [12].

Landmark detection can be performed manually or automatically. Manual landmark detection depends on medical expertise [13]. In addition to the necessity of previous experience, the process is time-consuming and tedious [14]. Hence, there have been efforts to computerize and automate cephalometric analysis based on 2D data [13] and 3D data [5,14]. For 2D images, four approaches are available when designing software that can locate cephalometric landmarks (image filtering plus knowledge-based landmark search, model-based approaches, soft-computing approaches, hybrid approaches). Leonardi et al. concluded that the errors in landmark detection using these methods were greater than those experienced with manual tracing, concluding that these systems are not accurate enough for clinical purposes [15].

To the best of our knowledge, few studies have focused on 3D data. Those that are available were limited to studying 3D surface-rendered models. For example, Mestiri and Hamrouni designed software using Reeb graphs [5]. Pan Zheng et al. used a Visualization Toolkit (VTK) and wrapper language Tcl/tk as a computer-assisted method. They suggested that automatic localization of 3D craniofacial landmarks should be studied in the future [14].

Although there are problems with manual detection, developing a fully automated system for identifying landmarks remains challenging [14]. We have found no published studies that have attempted to develop software that can detect landmarks based on image registration. Presumably, such an automated system would result in more convenient and accurate measurements. The present study was designed to produce software capable of automated localization of craniofacial landmarks based on image registration. We then evaluated its accuracy.

## Methods

### Study design

A total of 28 CBCT images were imported into our newly designed software. After three experts localized 14 landmarks, 8 of the 28 CBCT images were used as reference images to create a head atlas. The remaining 20 CBCT images were used as test images to evaluate our software.

This study was performed in accordance with the Declaration of Helsinki and was approved by the ethics committee of Shiraz University of Medical Sciences (ECSUMS) issued on 07 July 2013 (reference code EC-P-92-5479).

Written informed consent was obtained from each patient for the publication of this report and any accompanying images.

### Subjects

To create the atlas, 8 CBCT images that came from patients with ideal cephalometric measurements (ages 10–45 years, distributed in intervals of about 5 years) were selected from an archive of 500 previously acquired images at one of the two main private oral and maxillofacial radiology centers in Shiraz.

For testing our system, 20 CBCT images were randomly selected from the same archive. They were randomized using a random numbers table. The number selected from the table showed the CBCT code that would be selected from the archive. The inclusion criteria for test images were large field-of-view (FOV) CBCT images of orthodontic patients. Exclusion criteria included images with significant fractures or severe skeletal anomalies. All of the subjects were aged between 10 and 43 years.

Each patient was positioned in the NewTom VGi Cone Beam CT machine (QR SRL Company, Verona, Italy) with the aid of a guide light. The Frankfort horizontal plane (FHP) was parallel to the floor, and the mid-sagittal plane passed through the glabella. The examinations were performed at 4.71 mA and 110 kVp, with a scan time of 3.6 s. The FOV was 15 × 15 cm for all images.

The raw images from the CBCT scan were converted to digital information and communication in medicine (DICOM) three multifiles using the NNT viewer software version 2.21 (Quantitative Radiology, Verona, Italy). The DICOM images were loaded into our new software for localizing the landmarks manually and then automatically.

### Software

Technical experts designed the software using MATLAB programming language. This system is capable of presenting images that allow us to locate landmarks manually, reporting the coordinates of landmarks in x, y, and z planes, and detecting landmarks in its automated mode.

### Localizing landmarks manually

A total of 14 cephalometric landmarks were located based on the description of the landmarks provided by Zamora et al. [1]. The 14 landmarks were at point A, point B, anterior nasal spine (ANS), posterior nasal spine (PNS), pogonion (Pog), nasion (N), sella (S), gnathion (Gn), menton (Me), right gonion (Go), tip of the right upper central incisor (U1T), apex of the right upper central incisor (U1A), tip of the right lower central incisor (L1T), and apex of the right lower central incisor (L1A).

**Figure 1:**
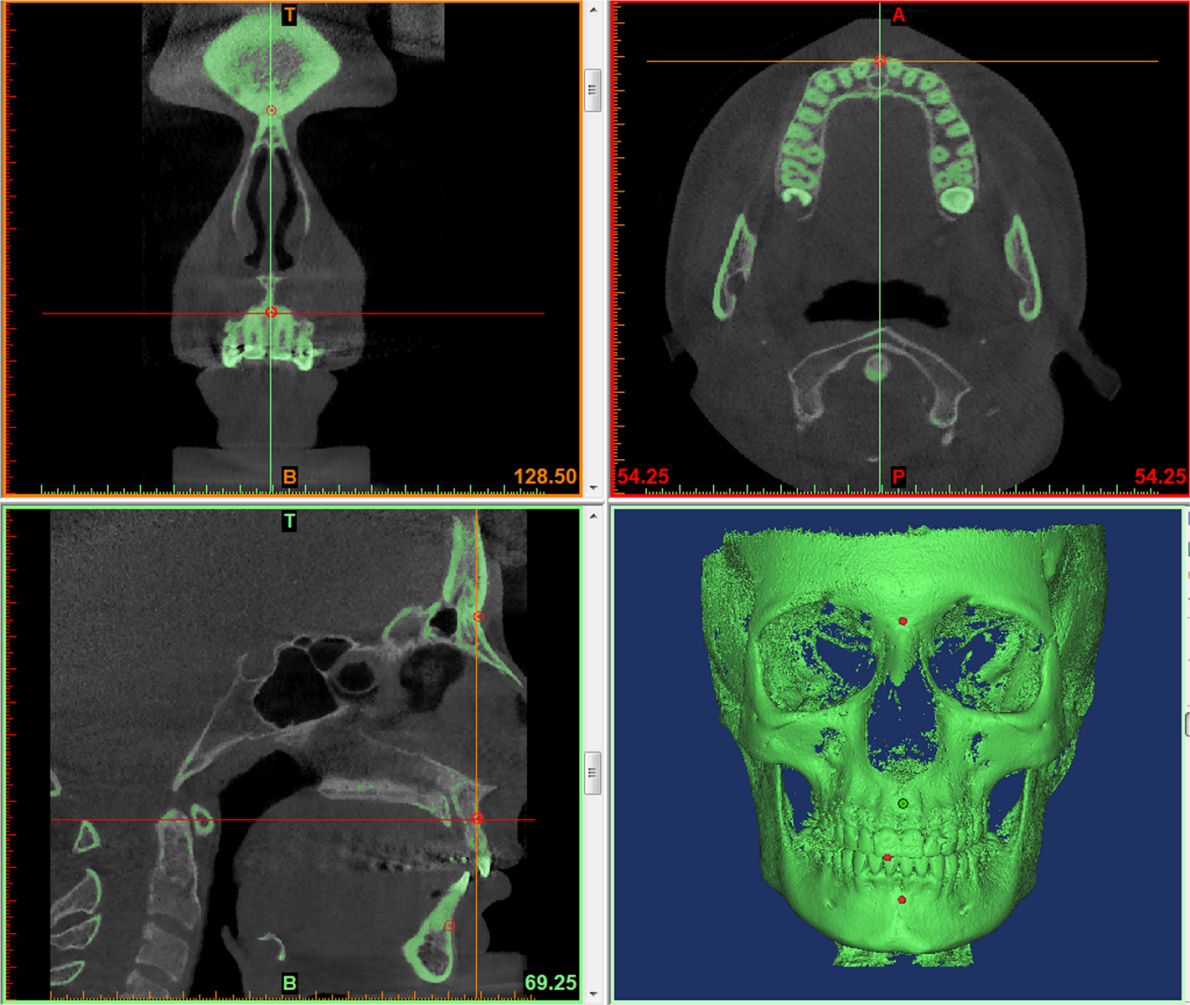
Landmark localization window displaying coronal, sagittal, and axial MPR views beside the 3D surface rendered skeletal volume.

Three experts (two orthodontists and one maxillofacial radiologist) were trained to locate landmarks using a set of three CBCT images not included in this study. Working independently after calibration and using the software, each of the three experts located the landmarks twice in all 28 CBCT images during two examinations separated by an interval of 6 weeks. Image presentation was in a four-panel window containing axial, coronal, and sagittal slices beside the 3D rendered model (Figure 1). Image enhancement features such as zoom in/out and changes of brightness and contrast were available for finding the landmarks more accurately. Landmarks were identified using a mouse-driven graphical cursor on the displayed 3D surface-rendered model, followed by adjusting the landmark using multiplanar reconstruction (MPR) images. This method was described by Bassam Hassan as the most precise method for localizing craniofacial landmarks [9]. The mean of the coordinates of six manual identifications of each landmark was defined as the baseline.

### Creating a 3D cephalometric head atlas

We needed to create a 3D cephalometric head atlas that contained the 14 normal landmarks as a reference model. For accurate registration, it was better to reduce the scaling, translation, and rotation among images. More similarity among the images would eliminate these factors, rendering the registration more accurate. We used each patient’s age as a criterion for the size of his or her head (older patients would have larger heads because of the natural growth process). We then used the eight CBCT images to create the atlas. When the software was presented with a test image, it automatically selected the reference image from among those eight samples based on age-matched data for the test subject and the reference subject.

### Automated landmark detection

**Figure 2:**
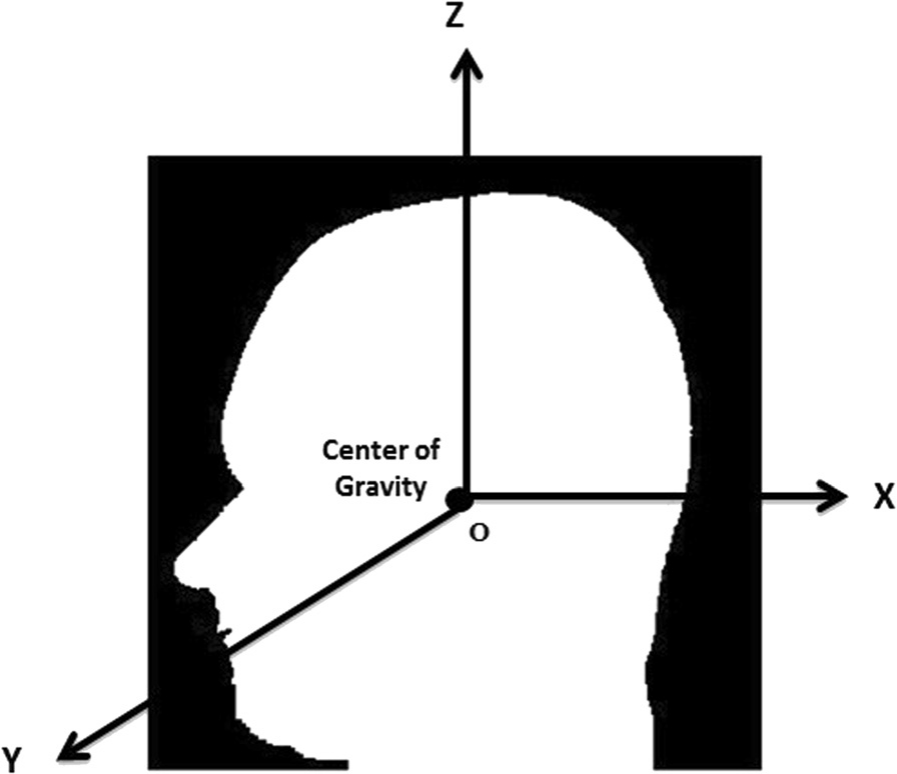
Schematic image showing centroid and principal axes.

**Figure 3:**
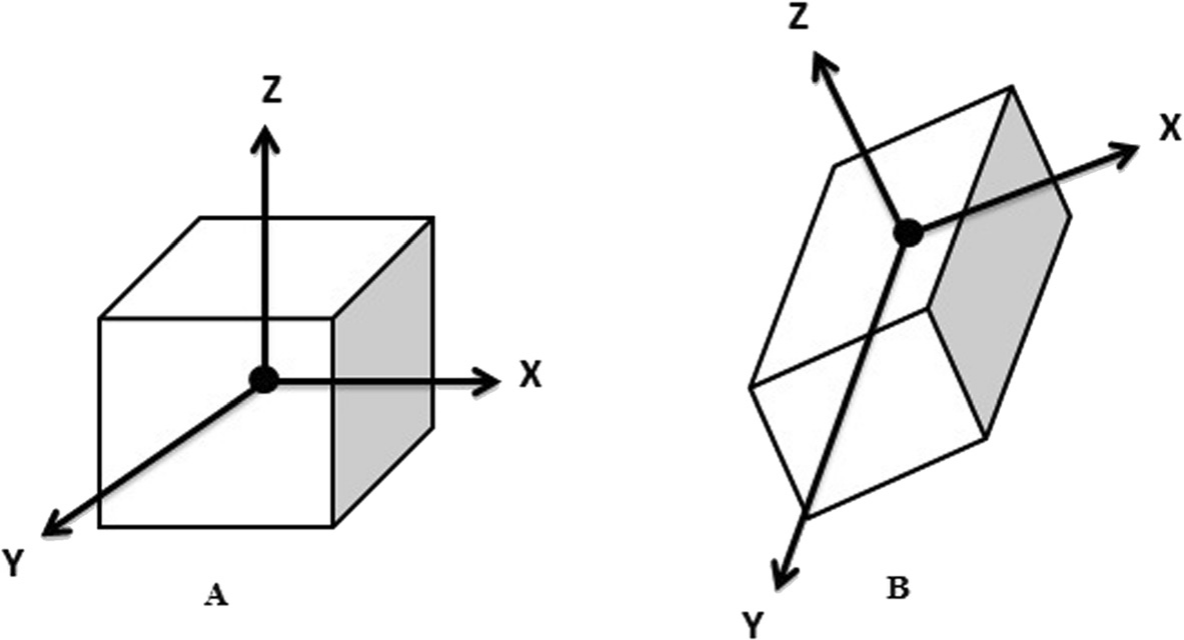
Schematic image showing scaling function and image registration, A is the reference image and B is the test image.

**Figure 4:**
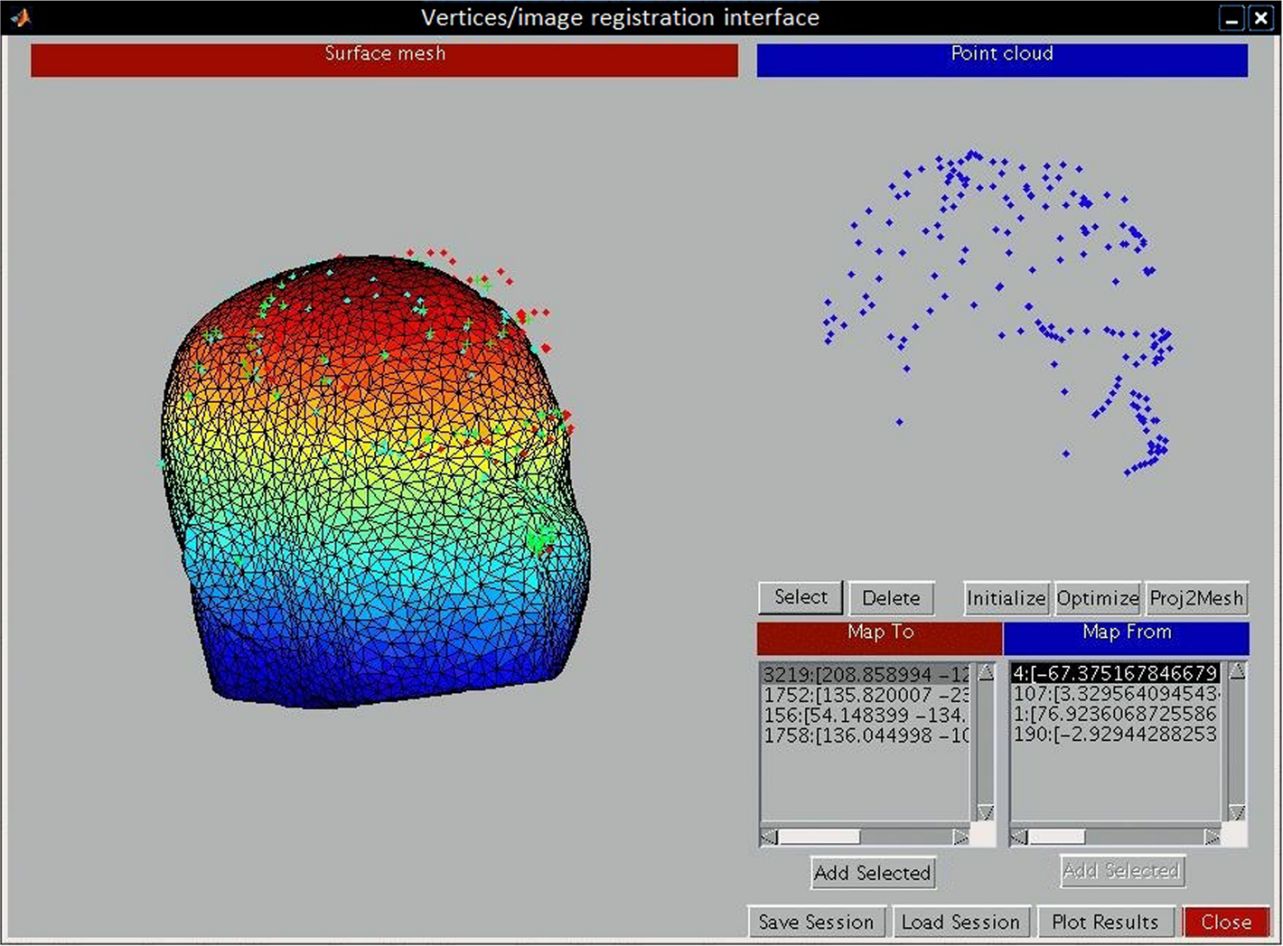
**Image registration interface.** The 3D image in the left side illustrating the test image and the blue points on the right side are the landmarks of the reference image used for registration process. These landmarks are different from the craniofacial landmarks.

**Figure 5:**
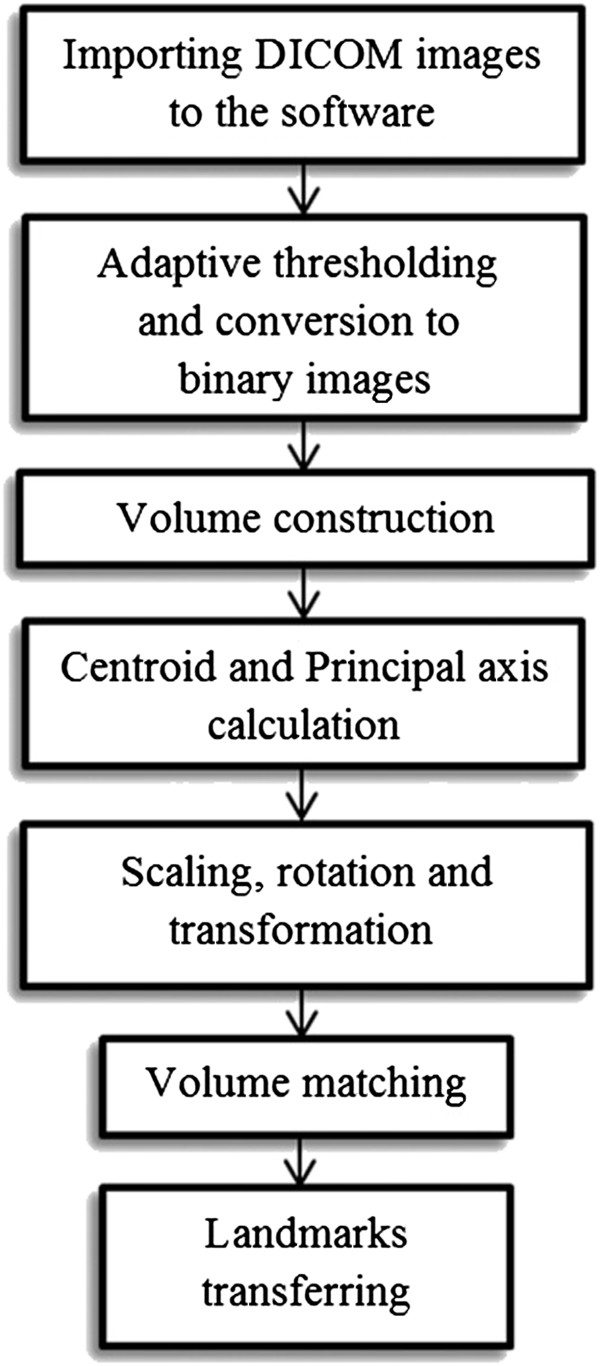
Flowchart of the proposed method.

We used image registration to transfer the landmarks from our atlas images to the test images. For this purpose, we used a method proposed by Too et al. that has the benefits of both feature-based and voxel similarity-based methods [16]. Briefly, transformation parameters are calculated based on the centroid and the principal axes of the two CBCT images (Figure 2). The method uses the whole 3D volume to provide reliable registration results. Extraction of the principal axis of the 3D object is accomplished in three steps: feature extraction, centroid calculation, and principal axis calculation. A binary volume is used as the feature to represent the 3D geometric shape to extract the principal axes. Gray-scale CBCT images were changed to binary images using adaptive thresholding. During this process, a threshold is calculated for each pixel in the image. The output is thus a binary image representing the segmentation. Principal axes and the centroids for both images were computed with respect to equations explained by Too et al. [16]. We completed the registration by scaling, rotation, and translation of the test image (Figures 3 and 4). After scaling, the test image vector was as long as the reference image vector in all directions (axial, coronal, sagittal). For the last step, the landmark was transferred between images. The flowchart of our algorithm is shown in Figure 5.

### Evaluation of the software

To evaluate the accuracy of the software regarding its automated detection of landmarks, we compared each landmark’s coordinates generated from the 20 CBCT images by the software to the mean values that experts had determined previously as the reference.

### Statistical analysis

All variables and measurements were introduced into a version 12.0 Excel spreadsheet (Microsoft Corp., Redmond, WA, USA) and were then analyzed using version 20.0 of the statistical package SPSS for Windows (IBM Corp., Somers, NY, USA). Intraclass correlation coefficients (ICCs) were used to determine intraobserver and interobserver agreement. To compare the values generated by the software to the values provided by experts, distances of coordinates of each landmark on each image between manual and automated detection methods were calculated with a 3D Euclidian distance formula,

$$\text{Distance}\;=\sqrt{{\left(x_{1}-x_{2}\right)}^{2}+{\left(y_{1}-y_{2}\right)}^{2}+{\left(z_{1}-z_{2}\right)}^{2}}\text{,}$$

where *x*_1_, *y*_1,_*z*_1_ are coordinates for manual detection and *x*_2_, *y*_2,_*z*_2_ are coordinates for automated detection.

## Results

The ICCs and 95% confidence intervals (95% CI) for intraobserver reliability on measurements were 0.86 (0.81 to 0.95) for observer 1; 0.89 (0.83 to 0.96) for observer 2; 0.93 (0.88 to 0.96) for observer 3. The combined ICC for intraobserver reliability was 0.89. The ICC and 95% CI for interobserver reliability were 0.87 and 0.82–0.93.

The minimum error, maximum error, mean error, standard error of the mean, and percentage of cases with <3 mm mean error from the baselines for the manually and automatically identified landmarks are summarized in Tables 1 and 2. According to Table 1, the most precisely identified landmark was the pogonion, with a 3 mm mean error. The least precisely identified landmark was point B, which had a mean error of 3.86 mm.

**Table 1:** The Min. error, Max. error, mean error and the standard error of the mean for the 6 manually detected landmarks’ coordinates from the mean of those coordinates as the gold standard

***Landmark***	***Min. error (mm)***	***Max. error (mm)***	***Mean error (mm)***	***Standard error of the mean (mm)***	***Percentage of cases with <3 mm mean error***
**A**	0.89	1.94	1.72	0.62	100%
**B**	0.95	1.85	1.36	0.52	100%
**ANS**	1.15	1.90	1.66	0.46	100%
**PNS**	1.63	2.35	2.10	0.38	100%
**Pog**	1.05	1.84	1.70	0.36	100%
**N**	0.96	1.80	1.64	0.48	100%
**S**	0.75	1.45	1.26	0.39	100%
**Gn**	1.05	2.60	2.15	0.89	100%
**Me**	0.86	1.40	1.24	0.26	100%
**Go**	1.22	2.54	1.96	0.66	100%
**U1T**	0.65	1.10	0.93	0.28	100%
**U1A**	0.54	0.89	0.74	0.22	100%
**L1T**	0.56	0.80	0.68	0.18	100%
**L1A**	0.45	0.75	0.59	0.19	100%
**Total**	0.45 for Point L1A	2.60 for Point Gn	1.41 mm	-	100%

**Table 2:** The Min. error, Max. error, mean error and the standard error of the mean for the automatically identified landmarks in millimeters from the baselines

***Landmark***	***Min. error (mm)***	***Max. error (mm)***	***Mean error (mm)***	***Standard error of the mean (mm)***	***Percentage of cases with <3 mm mean error***
**A**	1.58	4.77	3.11	0.74	60%
**B**	2.48	5.92	3.86	1.41	55%
**ANS**	2.38	4.52	3.12	0.80	70%
**PNS**	2.35	5.36	3.60	1.35	60%
**Pog**	2.02	4.78	3.00	1.02	60%
**N**	1.62	5.97	3.20	1.64	65%
**S**	1.83	6.12	3.45	1.82	55%
**Gn**	1.76	7.10	3.77	2.69	65%
**Me**	2.24	6.78	3.59	1.79	75%
**Go**	2.15	5.91	3.72	1.67	70%
**U1T**	2.22	6.70	3.59	1.76	70%
**U1A**	2.26	4.81	3.15	0.91	60%
**L1T**	2.31	4.84	3.30	0.92	60%
**L1A**	1.98	4.98	3.08	1.08	65%
**Total**	1.58 mm for Point A	7.10 mm for Point Gn	3.40 mm	-	63.57%

## Discussion

This study was performed to assess the accuracy of new software that had been specifically designed based on image registration. Its purpose was automated localization of craniofacial landmarks. Our approach was based on volume matching using the whole 3D volume. The mean errors of all 14 landmarks were <4 mm. Also, 63.57% of the landmarks had a mean error of <3 mm compared to manual detection (gold standard method). The mean error for all of the automatically identified landmarks in our study was higher than the mean error for the manually detected landmarks. However, in some studies on 2D images, a distance of ≤4 mm was considered acceptable [17,18]. De Oliveira et al. stated that the clinical significance of the accuracy of the landmark identification error depends on the level of accuracy required [12]. However, it seems that the acceptability of this error should be further evaluated. Mestiri and Hamrouni proposed a system that was designed to use Reeb graphs for automatic localization of cephalometric landmarks. The authors reported that 18 of 20 landmarks related to just one case were recognized successfully, with errors from baseline of 0.5 to 2.8 mm [5]. Pan Zheng et al. tried to visualize craniofacial landmarks and identify them using a Visualization Toolkit (VTK) and wrapper language Tcl/tk. Their method was not completely automated [14].

In this study, we used 28 CBCT images from an archive of 500 CBCTs. All the subjects had been positioned in the CBCT machine with the FHP parallel to the floor and the mid-sagittal plane passing through the glabella. Bassam Hassan et al. considered that 3D images were preferred to 2D images because of the higher accuracy they offered in regard to head position. They stated that small variations in the patient’s head position do not influence the measurement accuracy in 3D images, whereas there was a significant difference between the ideal and rotated scan positions for the 2D images [6].

In our study, three experts manually identified all landmarks in our 28 CBCT images at two separate sessions, ensuring the validity of the coordinates against our gold standard values. We asked them to use 3D surface-rendered models in addition to MPR images because the addition of MPR images to the 3D model can increase the precision of landmark detection. Research performed in 2009 showed that the use of MPR images takes full advantage of the 3D CBCT information, whereas locating landmarks on the 3D renderings alone can lead to error [12]. Bassam Hassan et al. in 2009 stated that performing cephalometric analysis on 3D-rendered models seems to be the most appropriate approach with regard to accuracy and convenience [6]. However, in research published in 2011, Bassam Hassan et al. concluded that addition of MPR images to the 3D model does have a positive influence on precision—but on average takes twice as much time [9].

The head atlas we created was uniquely designed to provide reference images for image registration. It was based on eight CBCT images from subjects at 5-year age intervals. All of the images were evaluated by experts using the 3D + MPR method, which ensures additional precision. Mestiri and Hamrouni stated that they had created a head atlas assisted by medical staff, but they did not explain their exact method [5].

A total of 20 CBCT images were used to evaluate the accuracy of our method. This number was considered suitable by the authors. In a systematic review, however, the number of images required to appraise the effectiveness of automated landmark detection in 2D images ranged from 5 to 600 [15]. We could not find similar studies on 3D images that reported such a considerable sample size. In a study performed at the University of El-Manar, only one CT image was used to evaluate the accuracy of their method (Reeb graph) for automated localization of the landmarks. Those authors stated that their method needed to be validated on a larger database [5]. Although we used more images in our study, we recommend repetition of similar studies using a larger sample size.

We used a new method for registration, first proposed by Too et al. They combined the advantages of feature-based and voxel similarity-based methods by converting the volumetric data to a binary volume as a feature, followed by use of Principal Axes Registration (PAR) [19]. We believed PAR to be the most effective one among the feature-based methods because it can find the transformation parameters easily and has less computational complexity [16]. Alpert et al. stated that registration by the principal axes transformation can be accomplished with typical errors in the area of ~1 mm. It also has the advantages of simplicity and speed of computation [19]. To the best of our knowledge, our study is the first conducted to implement and evaluate this method for automated landmark localization on maxillofacial CBCT images.

Although the current study presents a valuable method for automated detection of craniofacial landmarks on CBCT images, it has some limitations. First, we did not exclude images of patients with orthodontic braces. These images were responsible for most of our observed error, which reflects the impact of streak artifacts. We concluded that more errors occur with images of patients who have orthodontic appliances and probably surgical rigid fixation as well.

The radiation dosage in CBCT is another limitation that may limit implementation of this technique. The use of large-volume (craniofacial) CBCT imaging—i.e., the entire facial skeleton—is a common procedure for orthodontic-related radiological assessment by some clinicians [20,21]. According to the SEDENTEXTC guideline, however, its radiation dose, particularly in pediatric patients, is a challenging issue. The use of large volume CBCT may be justified when planning a definitive procedure in complex cases of skeletal abnormality, particularly those requiring combined orthodontic/surgical management [22]. However, it is not plausible to expose all orthodontic patients to the radiologic dose of CBCT with the intention of cephalometric analysis per se.

The database used in the present study included only Iranian patients. Therefore, our results may not be applicable to other populations. We suggest that further research be undertaken using appropriate databases from other ethnic groups. We also suggest that image registration using other approaches be attempted in the future. Intensity-based methods seem to be accurate and suitable for clinical application [23]. We do recommend evaluations of measurement analyses based on automated landmark detection methods.

## Conclusion

We contend that this software is the first to use a combined method (feature-based and voxel similarity-based) for image registration during automated localization of craniofacial landmarks on CBCT images. The accuracy of our method was acceptable. Nevertheless we recommend repetition of this study using other techniques, such as intensity-based methods.

## Competing interests

The authors declare that they have no competing interests.

## Authors’ contributions

ShSh devised the study concept, designed the study, supervised the intervention, data collection and analysis, participated in the coordination of the study, and critically revised the manuscript. BE and SE collected data, ran the study intervention, participated in the study concept, performed the analyses, and drafted the manuscript. ZA participated in the intervention, data collection and revision of the manuscript. OM contributed to the design and analysis of the study data, and revised the manuscript. MM participated in the intervention, data collection and revision of the manuscript. MA contributed to the design and intervention of the study, and manuscript revision. All authors read and approved the final manuscript.

## Pre-publication history

The pre-publication history for this paper can be accessed here:

http://www.biomedcentral.com/1471-2342/14/32/prepub

## References

[B1] Zamora N Llamas JM Cibrian R Gandia JL Paredes V Cephalometric measurements from 3D reconstructed images compared with conventional 2D images Angle Orthod 2011 81 856 864 10.2319/121210-717.1 21469969 PMC8916191

[B2] Miller SBDM Computer-aided head film analysis: the University of California San Francisco method Am J Orthod 1980 78 41 65 10.1016/0002-9416(80)90039-1 6930171

[B3] Forsyth DB Shaw WC Richmond S Roberts CT Digital imaging of cephalometric radiographs, Part 2: Image quality Angle Orthod 1996 66 43 50 8678345 10.1043/0003-3219(1996)066<0043:DIOCRP>2.3.CO;2

[B4] Ludlow JB Gubler M Cevidanes L Mol A Precision of cephalometric landmark identification: cone-beam computed tomography vs conventional cephalometric views Am J Orthod Dentofacial Orthop 2009 136 312 e311-310; discussion 312-313 10.1016/j.ajodo.2009.04.009 19732656 PMC2753840

[B5] Makram M Kamel H Reeb Graph for Automatic 3D Cephalometry IJIP 2014 8 17 29

[B6] Hassan B van der Stelt P Sanderink G Accuracy of three-dimensional measurements obtained from cone beam computed tomography surface-rendered images for cephalometric analysis: influence of patient scanning position Eur J Orthod 2009 31 129 134 10.1093/ejo/cjn088 19106265

[B7] Chen YJ Chen SK Yao JC Chang HF The effects of differences in landmark identification on the cephalometric meaurements in traditional versus digitized cephalometry Angle Orthod 2004 74 155 161 15132440 10.1043/0003-3219(2004)074<0155:TEODIL>2.0.CO;2

[B8] Houston WJB The analysis of errors in orthodontic measurements Am 1 Orthod 1983 83 http://www.ncbi.nlm.nih.gov/pubmed/?term=Houston+WJB%3A+The+analysis+of+errors+in+orthodontic+measurements+Am+1+Orthod+1983%2C+83 10.1016/0002-9416(83)90322-6 6573846

[B9] Bassam H Peter N Hans V Jamshed T Christian V van der Stelt P Beek H Percision of identifying cephalometric landmarks with cone beam computed tomography in vivo Euro J Orthodontics 2011 doi:10.1093/ejo/cjr050 10.1093/ejo/cjr050 21447781

[B10] Gribel BF Gribel MN Manzi FR Brooks SL McNamara JA Jr From 2D to 3D: an algorithm to derive normal values for 3-dimensional computerized assessment Angle Orthod 2011 81 3 10 10.2319/032910-173.1 20936948 PMC8926363

[B11] Couceiro CP Vilella OV 2D/3D Cone-Beam CT images or convensional radiography: Which is more reliable? Dental Press J Orthod 2010 15 40 41 10.1590/S2176-94512010000500007

[B12] de Oliveira AE Cevidanes LH Phillips C Motta A Burke B Tyndall D Observer reliability of three-dimensional cephalometric landmark identification on cone-beam computerized tomography Oral Surg Oral Med Oral Pathol Oral Radiol Endod 2009 107 256 265 10.1016/j.tripleo.2008.05.039 18718796 PMC2642991

[B13] Shahidi S Oshagh M Gozin F Salehi P Danaei S Accuracy of computerized automatic identification of cephalometric landmarks by a designed software Dentomaxillofac Radiol 2013 42 20110187 10.1259/dmfr.20110187 23236215 PMC3746488

[B14] Pan Z Bahari B Rozniza Z Arash I Rajion ZA Computerized 3D Craniofacial Landmark Identification and Analysis eJCSIT 2009 1

[B15] Rosalia L Daniela G Francesco M Spampinato C Automatic Cephalometric Analysis A Systematic Review Angle Orthod 2008 78 145 10.2319/120506-491.1 18193970

[B16] Naw Chit TJ Xuenan C Shengzhe L Hakil K Fast and Accurate Rigid Registration of 3D CT Images by Combining Feature and Intensity J Comp Sci Eng 2012 6 1 11 10.5626/JCSE.2012.6.1.1

[B17] Yue W Yin D Li C Wang G Xu T Automated 2-D cephalometric analysis on X-ray images by a model-based approach IEEE Trans Biomed Eng 2006 53 1615 1623 16916096 10.1109/TBME.2006.876638

[B18] El-Feghi IS-AM Ahmadi M Automatic localization of craniofacial landmarks for assisted cephalometry Pattern Recognit 2004 37 609 621 10.1016/j.patcog.2003.09.002

[B19] Alpert NM Bradshaw JF Kennedy D Correia JA The principal axes transformation–a method for image registration J Nuc Med 1990 31 1717 1722 2213197

[B20] Kapila S Conley RS Harrell WE Jr The current status of cone beam computed tomography imaging in orthodontics Dentomaxillofac Radiol 2011 40 24 34 10.1259/dmfr/12615645 21159912 PMC3611465

[B21] Smith BRPJ Cederberg RA An evaluation of cone-beam computed tomography use in postgraduate orthodontic programs in the United States and Canada J Dent Educ 2011 75 98 106 21205734

[B22] Radiation protection: cone beam CT for dental and maxillofacial radiology. Evidence based guidelines 2011 SEDENTEXCT Project [ http://www.sedentexct.eu/files/guidelines_final.pdf]

[B23] Hill DL Studholme C Hawkes DJ "Voxel similarity measures for automated image registration," Visualization in Biomedical Computing 1994 Bellingham, WA: SPIE 205

